# Supplementary material for: Exergy Analysis of a Bio-System: Soil–Plant Interaction
Source: Entropy (Basel). 2020 Dec 23;23(1):3. doi: 10.3390/e23010003 (PMC7822430; doi:10.3390/e23010003)
Supplement: Supplementary file 1 [file entropy-23-00003-s001.pdf]

entropy MDPI

Photosynthesis includes two main processes as PSII and PSI. The process is underpinned by the light-driven water splitting reaction that occurs in PSII of plants, algae, and cyanobacteria. Solar energy is absorbed by chlorophyll and other pigments and is transferred efficiently to the PSII reaction center where charge separation takes place. This initial conversion of light energy into electrochemical potential occurs in the reaction center of PSII with a maximum thermodynamic efficiency of about 70%. In principle, it could drive the formation of hydrogen. Instead, the reducing equivalent is passed along an electron transport chain to PSI, where it is excited by the energy of a second 'red' photon absorbed by a chlorophyll molecule. In this way, sufficient energy is accumulated to drive the fixation of carbon dioxide, which not only requires the generation of the reduced 'hydrogen carrier', nicotinamide adenine dinucleotide phosphate (NADPH) but also the energy-rich molecule adenosine triphosphate (ATP) formed by the release of some energy during electron transfer from PSII to PSI (in the form of an electrochemical potential gradient of protons)[1].

In the Calvin cycle, carbon atoms from CO_2 are fixed (incorporated into organic molecules) and used to build three-carbon sugars. This process is fueled by, and dependent on, ATP and NADPH from the light reactions. Unlike the light reactions, which take place in the thylakoid membrane, the reactions of the Calvin cycle take place in the stroma (the inner space of chloroplasts). This process is shown in Figure S 1 and Figure S 2.

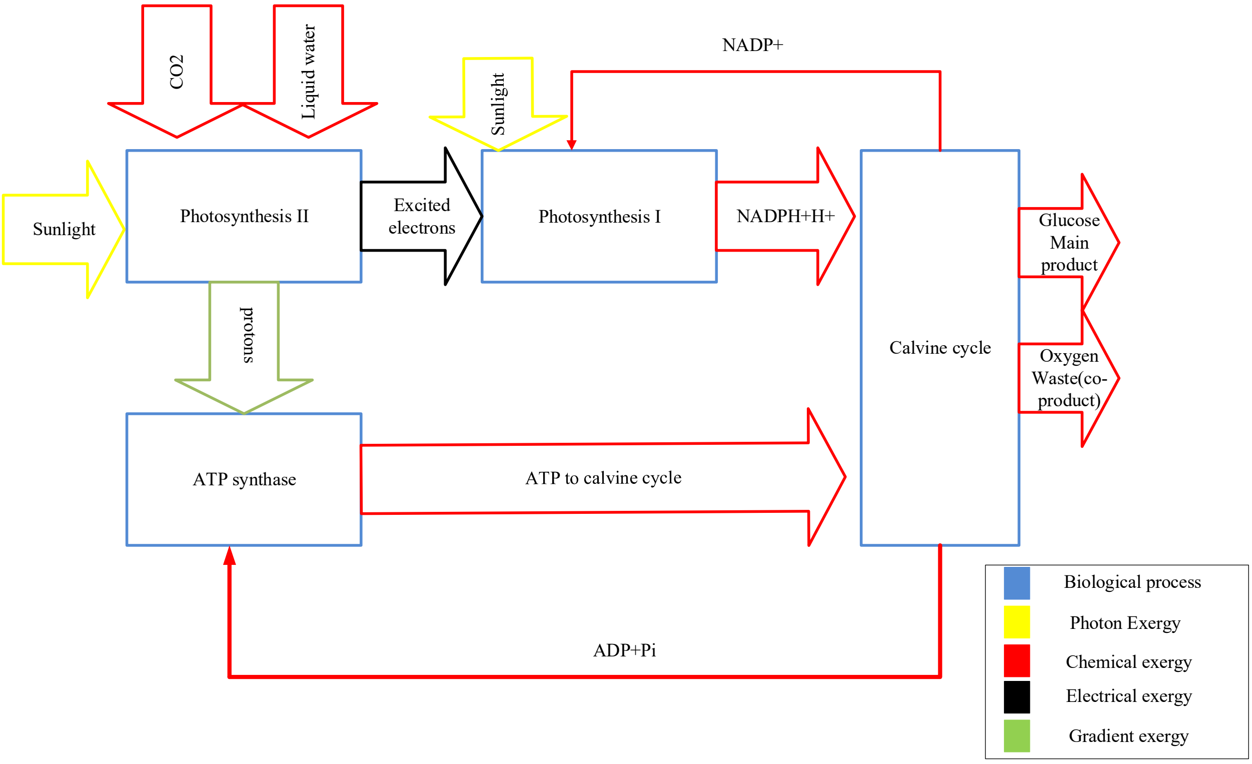

Figure S 1. Qualitative Exergy-Flow Diagram of the plant. The Color Key describes the type of exergy flows between the different biological operations[2]

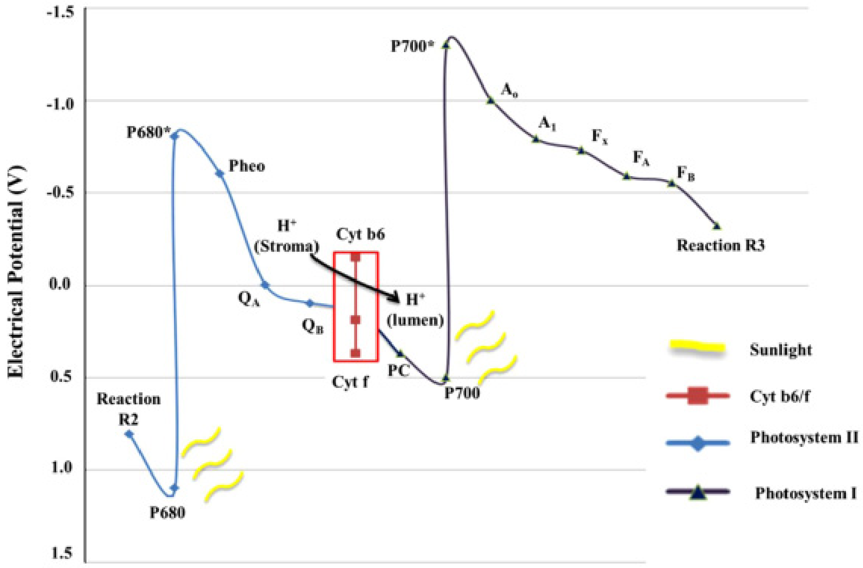

Figure S 2. Transfer of high energy electrons through photosystem II (PSII) and photosystem I (PSI)[3]. Two chemical reactions are described in this figure. All of the reactions are explained here. In the first step, water is split into protons, oxygen, and electrons. The electrons are excited to the high-level energy (P680*). NADPþ is reduced to NADPH. Intermediate carriers are various functional groups in the protein complexes of PSII and PSI.

The first assumption of the model is based on Table S 1:

Table S 1. The assumption for the average photon exergy

Parameter	Unit	Value
Avogadro's number (NA)		6.023E+23
Planck's constant (h)	Js	6.626E-34
speed of ligh (c)	m/s	300000000
λ_high(nm)	nm	700
λ_low	nm	400
T_earth(K)	K	298.15
T_sun	K	5762
B_photon_ave(kJ/mol photon)	kJ/mol photon	212

Table S 2, Table S 3, Table S 4, and Table S 5 are the exergy analysis for photosynthesis and the Calvin cycle processes.

Table S 2. Exergies and reduction potentials of PSII

Reactions	Redox potential (V)	standard Gibbs free energy change(kJ/mol)	standard change in electrical potential (V)	Exergy change (J)
$2H_2O \rightarrow O_2 + 4H^+$	0.81			
P680	1.1	-0.29	-671536	-819489

P680(required exergy from sun)	-0.8	1.9	4399716	4399716
Pheo	-0.6	-0.2	-463128	-463128
Qa	0	-0.6	-1389384	-1389384
Qb	0.1	-0.1	-231564	-231564
Cytb	0.19	-0.09	-208408	-208408
PC	0.37	-0.18	-416815	-416815
total differernce	0.37	-0.44	1018882	870928

Table S 3. Exergies and reduction potentials of PS

**Reactions**	**Redox potential (V)**	**standard Gibbs free energy change (kJ/mol)**	**standard change in electrical potential (V)**	**Exergy change (J)**
PC	0.37			
P700	0.5	0.13	-301033	-301033
P700(required exergy from sun)	-1.3	-1.8	4168152	4168152
A0	-1.3	-1.8	-694692	-694692
A1	-1	0.3	-486284	-486284
Fx	-0.79	0.21	-138938	-138938
Fa	-0.73	0.06	-324190	-324190
Fb	-0.59	0.14	-92626	-92626
Fd	-0.55	0.04	-46313	-46313
NADPH	-0.53	0.02	-486284	-486284
total difference	-0.32	0.21	1597792	1597792
total difference(NADPH -H2O)	-0.32	-1.13	2616173	2468720

Table S 4. Exergy losses in the dark reactions

**Calvin Cycle reactions**	**total exergy difference(δB(J))**
$C1: (Ru5P) + (ATP) \rightarrow (RuBP) + (ADP)$	98582
$C2: CO_2 + (RuBP) + H_2O \rightarrow 2 \times (PGA)$	322242
$C3 + C4: (PGA) + (ATP) + (NADPH) \rightarrow (ADP) + (GAl3P) + (NADPH^{+}) + H_3PO_4$	32746
$C5: (GAl3P) \rightarrow (DHAP)$	755
$C6: (GAl3P) + (DHAP) \rightarrow (FBP)$	1974
$C7: (FBP) + H_2O \rightarrow (F6P) + H_3PO_4$	57933
$C8: (F6P) + (GAl3P) \rightarrow (E4P) + (Xu5P)$	6035
$C9: (E4P) + (DHAP) \rightarrow (SBP)$	2023
$C10: (S7P) + (GAl3P) \rightarrow (S7P) + H_3PO_4$	62498
$C11: (S7P) + (GAl3P) \rightarrow (R5P) + (Xu5P)$	11187
$C12: (R5P) \rightarrow (Ru5P)$	1289
$C13: (Xu5P) \rightarrow (Ru5P)$	766

SUM	59803
conversion to glucose	total exergy difference(δB(J))
$C5^*: (GAl3P) \rightarrow (DHAP)$	189
$C6^*: (DHAP) + (GAl3P) \rightarrow (FBP)$	987
$C7^*: (FBP) + H_2O \rightarrow (F6P) + H_3PO_4$	28966
$C14: (F6P) \rightarrow (G6P)$	1298
$C15: (G6P) + H_2O \rightarrow (Glucose) + H_3PO_4$	31768
conversion to glucose SUM	63208
TOTAL SUM	661239

Table S 5. Overall chloroplast efficiency

**Reactions**	**Inlet (kJ)**	**Outlet (kJ)**	**Loss (kJ)**	**Efficiency**	**Overall Loss(%)**	**PAR Loss(%)**
PAR Reflection	9977	9977	0	1	0	0
Non-PAR Reflection	13226	661	12564	0.05	61.33	
Photosystem II absorption	5319	4193	1126	0.788	5.5	14.45
photosystem II ETC	5319	4074	1246	0.766	6.08	15.99
photosystem I ETC	4209	inlet 2200	2009	0.523	9.81	25.79
ATP synthase	4901	2401	2500	0.49	12.2	32.09
Calvin cycle(Dark reaction)	1372	992	381	0.722	1.86	4.89

References

[1] J. Barber, "Photosynthetic energy conversion: natural and artificial," *R. Soc. Chem. 2009*, vol. 38, no. 1, pp. 185–196 |, 2009, doi: 10.1039/b802262n.

[2] C. S. Silva, W. D. Seider, and N. Lior, "Exergy efficiency of plant photosynthesis," *Chem. Eng. Sci.*, vol. 130, pp. 151–171, 2015, doi: 10.1016/j.ces.2015.02.011.

[3] M. M. C. David L. Nelson, *Lehninger Principles of Biochemistry*, Fourth Edi. W. H. Freeman, 2004.
